# Lithium biofortification of medicinal tea *Apocynum venetum*

**DOI:** 10.1038/s41598-019-44623-3

**Published:** 2019-06-03

**Authors:** Li Jiang, Lei Wang, Mohsin Tanveer, Changyan Tian

**Affiliations:** 1 State Key Laboratory of Desert and Oasis Ecology, Xinjiang Institute of Ecology and Geography, Chinese Academy of Sciences, Urumqi, 830011 China; 2 Key Laboratory of Biogeography and Bioresource in Arid Land, Xinjiang Institute of Ecology and Geography, Chinese Academy of Sciences, Urumqi, 830011 China; 3 School of Land and Food, University of Tasmania, Hobart, Australia

**Keywords:** Plant ecology, Nutrition

## Abstract

Lithium (Li) could be much safer and successful approach to supply Li via Li-fortified food products. This study is highlighting the potential scope of Li supply via Li-biofortification of Luobuma tea (made from *Apocynum venetum* leaves), which is a very popular beverage in Asia with several medical properties. We explored the possibility of *A. venetum* as Li-enriched tea and investigated plant growth, Li accumulation, total flavonoids (TFs), rutin and hyperoside concentrations, and the antioxidant capacity of *A. venetum*. With the increase of additional Li, Li concentration in roots, stems and leaves increased gradually. Compared with the control treatment, 10–15 mg kg^−1^ Li addition stimulated the growth of *A. venetum* and 25 mg kg^−1^ Li addition significantly increased the Li concentration in leaves by 80 mg kg^−1^. Li application did not decrease TFs, rutin, hyperoside and antioxidant capacity of this medicinal herb. A daily consumption of 10 g Li-biofortified *A. venetum* leaves (cultivated with 25 mg kg^−1^ LiCl) can give 592 μg Li intake and would constitute 59% of the provisional recommended dietary daily intake of Li. Our results showed that Li-biofortified *A. venetum* leaves can be used as Li-fortified tea to enhance Li supply and to improve human health when it was used as daily drink.

## Introduction

Lithium (Li) is increasingly regarded as an essential trace element for animals and humans^1^. Li deficiency could delay growth, short life expectancy, reduce milk performance and reproduction in goats^2^. Li also affects human behavior^3^. For example, there is a negative association between high Li concentration in drinking water and the incidences of homicides or suicides, as high lithium levels may influence impulsiveness levels and mediate the manifestation of aggressiveness^4^. Li is also used in pharmacological doses for the treatment of bipolar disorder^5^. Li plays a role in numerous biological processes such as enzymatic activity, channels activity and energy production in living organism^6–8^.

Li, the lightest of the alkali metals with high chemical activity, is relatively widespread in the environment and presents in trace amounts in soil (7–200 mg kg^−1^), mainly in clay fraction of soil and in surface water (1–10 μg L^−1^). Generally, the concentration of soil Li in arid and semiarid zone is relative high, especially in closed basins^7^. The amount of lithium present in plants depended on its abundance in the soil, soil pH and the type of plant^9,10^. Li is mainly supplied in relatively low doses with diet, particularly through consumption of grains and vegetables, and also with drinking water^7^. However, Li concentration in different food item varies with different natural environments. Foodstuffs may provide the following mean Li concentration (mg kg^−1^): cereals-4.4, fish-3.1, mushrooms-0.19, vegetables-2.3, meat-0.012, nuts-8.8 and dairy products-0.5. Though above food products can supply Li in our food chain however still there is no guarantee that these items will supply same amount of Li every time. Recently it has been suggested to consider an introduction of the food fortified with lithium similarly to the model of table salt iodization^3,11^. Moreover, cultivation of Li-fortified mushrooms has also been proposed however instant availability and safe storage of Li-fortified mushroom would be the biggest setback^12,13^. Therefore this study was conducted to examine; is there any possibility to provide/identify such food item (Li-fortified) which would be commercially cheap and instant available to public?

*Apocynum venetum* is a wild shrub widely distributed in northwestern China, especially in salt-barren zone, desert margins, alluvial flats, riversides^14^. Luobuma tea, made from leaves of *A. venetum*, is a popular daily beverage in China. *Apocynum venetum* is a potentially rich target of Li biofortification owing to its ability to accumulate Li in natural habitat^15,16^. This plant is used as traditional Chinese and Uygur medicine to treat hypertension, nephrosis, neurasthenia and hepatitis^14,17^. Some studies have shown that pharmacological propertes of *A. venetum* can be explained by the existence of various flavonoid compounds in leaves. However, the medicinal effects of *A. venetum* might also be attributed to the existence of high level of Li. Thus another objective of this study was to evaluate the feasibility of *A. venetum* for the bio-enrichment with Li. For this purpose, the study evaluated the biomass, Li accumulation and the potential effect that Li could have on the concentration of total flavonoids (TFs), rutin and hyperoside concentrations, and the antioxidant capacity. At the end, we also hypothesized that are Li and flavonoids concentrations and the antioxidant capacity in *A. venetum* leaves positively associated with Li application? The specific research questions were: which concentration of soil additional Li is optimal for plant growth? What is the relationship between concentrations of Li in *A. venetum* leaves and Li addition?

## Materials and Methods

### Pot experiment

The substrate used in this experiment was collected from Junggar desert (44°40′N, 87°86′E). This sandy soil was air-dried, tilled, homogenized and sieved through a 4-mm mesh to remove any debris material. The soil physical and chemical properties was weak alkaline (pH = 7.81) with organic matter of 0.90%, and contained total N-0.90 g kg^−1^, available N-81.45 mg kg^−1^, available P-8.98 mg kg^−1^, available K-335.26 mg kg^−1^, and Li 13.20 mg kg^−1^. Pot experiment was carried out in a greenhouse (28 ± 3 °C day/18 ± 3 °C night, natural light condition) at Fukang Field Research Station of the Chinese Academy of Sciences (44°17′N, 87°56′E), Xinjiang, China. Each pot (35 cm height; 33 cm diameter) contained 10 kg of soil mixed with different concentrations of Li (solution prepared by dissolving analytical grade LiCl (Fuchen Chemical Reagents, TianJin, China)): 0, 10, 15, 20, and 25 mg kg^−1^. To simulate the Li impacted soils, mixed soil in pot was homogenized for four weeks. Two *A. venetum* plants (rhizome cuttings, ca. 10 cm high) were transplanted into each pot. Six replicates were run for each treatment and arranged in a completely randomized design. For the fertilization treatment, each pot received 10 g Osmocote 301 with a 15N : 11P : 13K : 2Mg elemental ratio as the basic fertilizer. Pots were irrigated using distilled water every 2^nd^ day and the amount of water was estimated as 1 L per pot.

### Plant growth

Plant growth, Li and TFs concentrations were determined after 90 days of treatment. Leaves, stems and roots were separated and rinsed with distilled water three times, and then blotted with filter paper. After fresh material was dried at 80 °C for 48 h, the samples were weighed, ground, and sieved through 60-mesh.

### Li concentration

Dry sample (0.2 g) was digested with 10 ml of 50% HNO_3_ (v/v) in triangular flask overnight. Then, 3 ml of 30% H_2_O_2_ (v/v) was added and kept in a temperature control electric cooker (Lab Tech EH35A) at 150 °C for 10 min. After cooled to 20 °C, 5 ml of 1% HNO_3_ (v/v) and 1 ml of 30% H_2_O_2_ (v/v) were added. Samples were digested at 150 °C until the white smoke emitted fully, and the solution was transparent. After cooled to 20 °C, the solution was diluted to 50 ml with 1% HNO_3_ (v/v). Li concentration was estimated with an inductively coupled plasma spectroscopy (Perkin Elmer SciexDRC II) and determined at 670.783 nm.

### Total flavonoids (TFs)

TFs concentration in the crude extracts was determined by using the aluminum chloride colorimetric method of Chang *et al*. with some modifications^18^. 1 ml of the plant extract was mixed with 3 ml methanol, 0.2 ml of 10% aluminum chloride, 0.2 ml of 1 M potassium acetate and 5.6 ml of distilled water. Then the solution was incubated for 30 minutes at room temperature. The absorbance was measured at 415 nm using UV-visible spectrophotometer against a blank. The TFs concentration of plant methanolic extracts was expressed in Quercetin Equivalent.

### Rutin and hyperoside concentration

Rutin and hyperoside in leaves of *A. venetum* were determined by HPLC. The leaves were extracted with 60% ethanol by reflux extraction. The separation was carried out on an Agilent TC-C_18_ column (150 mm × 4.6 mm, 5 μm) eluted with the mobile phases of acetonitrile-0.1% phosphoric acid (16:84). The column temperature was 40 °C, and the flow rate was 1.0 mL min^−1^, the detection wavelength was 360 nm^19^.

### Antioxidant properties determined by DPPH and FRAP assay

Antioxidant capacity of common black tea (Jinjunmei, Fujian China), green tea (Biluochun, Fujian China) and leaves of *A. venetum* were determined. The plant material (0.5 g) was used for phenolic extraction with distilled water at 50 °C under agitation. There were three replicates for black and green tea, and five replicates for *A. venetum*.

For DPPH assay, samples were incubated for 20 min at 37 °C in a water bath, and then the decrease in absorbance was measured at 515 nm (AE). A blank sample containing 100 µL of methanol in the DPPH solution was prepared and its absorbance was measured (AB). Radical scavenging activity was calculated using the following formula: The scavenging rates of DPPH = [1 − AE/AB] × 100^20^.

For FRAP assay, The ferric reducing power of plant extracts was determined using a modified version of the FRAP assay^21^. The reaction mixture was incubated for 30 min at 37 °C in a water bath. The absorbance of the samples was measured at 593 nm. The difference between sample absorbance and blank absorbance was calculated and used to calculate the FRAP value. FRAP values were expressed as mmol g^−1^ Fe^2+^ of sample.

### Data analysis

All data were expressed as mean ± standard error (S.E.). All tests were performed with SPSS Version 16.0 for Windows. The correlation analysis was used to study the effect of soil Li concentration on Li concentration in leaves of *A. venetum* in the field. One-way analysis of variance (ANOVA) was used to compare treatment effects. Duncan’s test was used to test for differences among treatments when ANOVA showed significant effects (*P* < 0.05).

## Results

### Plant growth

**Figure 1:**
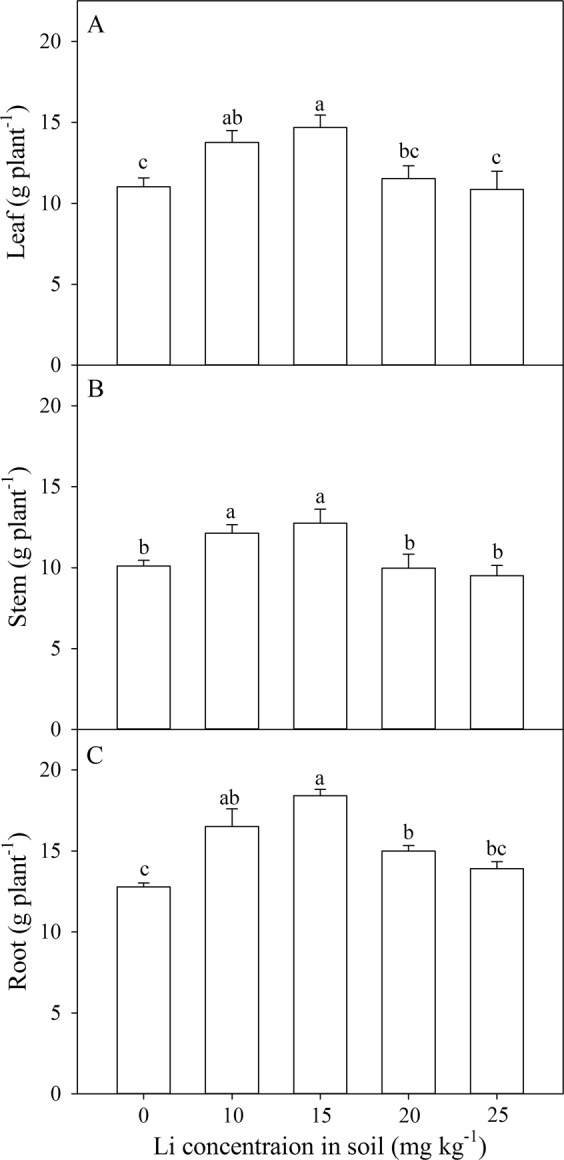
Effect of soil Li addition on leaf dry weight (**A**), stem dry weight (**B**), and root dry weight (**C**) of *A. venetum*. The values (mean ± S.E) share the same letters above the bars are not significantly different at P < 0.05. n = 6.

*A. venetum* showed varied biomass accumulation of different plant parts under different Li treatment (Fig. 1). Leaf dry weight, stem dry weight and root dry weight was increased by 24.97–33.38%, 20.14–26.24%, and 29.29–44.12% in response to 10 and 15 mg kg^−1^ soil Li addition respectively however these dry weight were not affected significantly by 20 mg kg^−1^ and 25 mg kg^−1^ soil Li addition. Our results clearly indicated that Li at low concentration enhanced plant biomass accumulation.

### Li concentration in different plant parts

**Figure 2:**
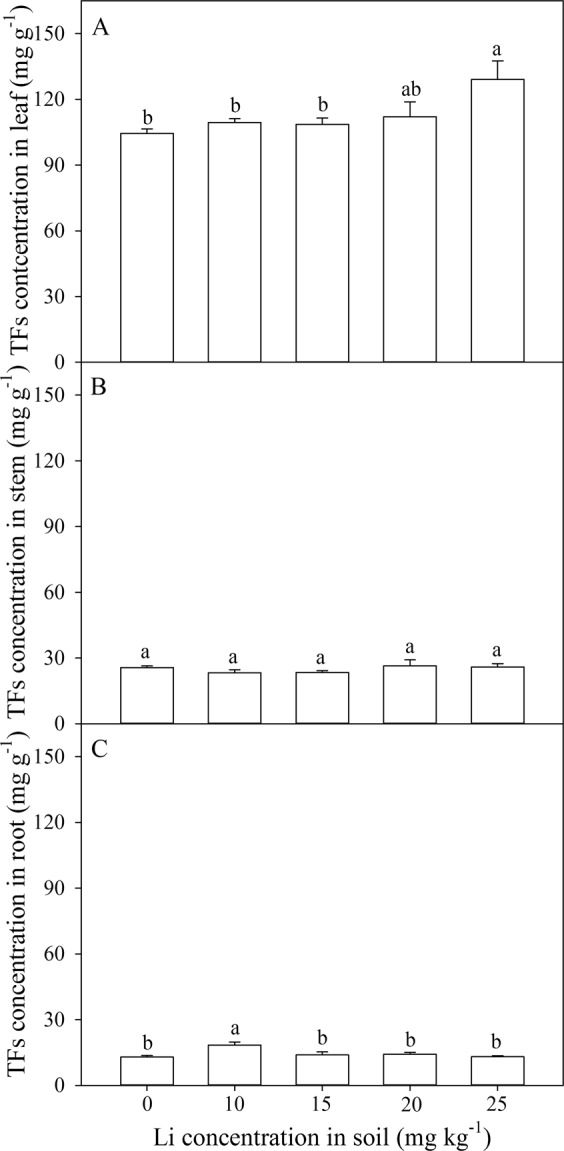
Effect of soil Li addition on the Li concentrations in leaf (**A**), stem (**B**), and root (**C**) of *A. venetum*. The values (mean ± S.E) share the same letters above the bars are not significantly different at P < 0.05. n = 6.

With the additional soil Li was raised, Li concentrations in leaves, stems and roots increased gradually. The regression equation of the relationship between Li concentrations in leaves and soil additional Li is: y = 0.0881x^2^ − 0.1074x + 16.873 (R^2^ = 0.97); The regression equation for Li concentrations in stems and soil additional Li is: y = 0.0839x^2^ − 0.1812x + 13.098 (R^2^ = 0.96); The regression equation for roots is: y = 0.0396x^2^ − 0.1365x + 10.461 (R^2^ = 0.88). When soil Li addition was 15 mg kg^−1^, Li concentrations in leaves, stems and roots were 15.60, 28.50, and 37.20 mg kg^−1^ respectively, with the increments in leaves, stems and roots were 1.19, 1.31, and 0.47 times, respectively. When additional Li was 25 mg kg^−1^, Li concentrations in leaves, stems and roots were 30.82, 62.76, and 73.98 mg kg^−1^ respectively with the increments in leaves, stems and roots were 3.36, 4.08, and 1.91 times, respectively (Fig. 2).

### Total flavonoids (TFs) concentration

**Figure 3:**
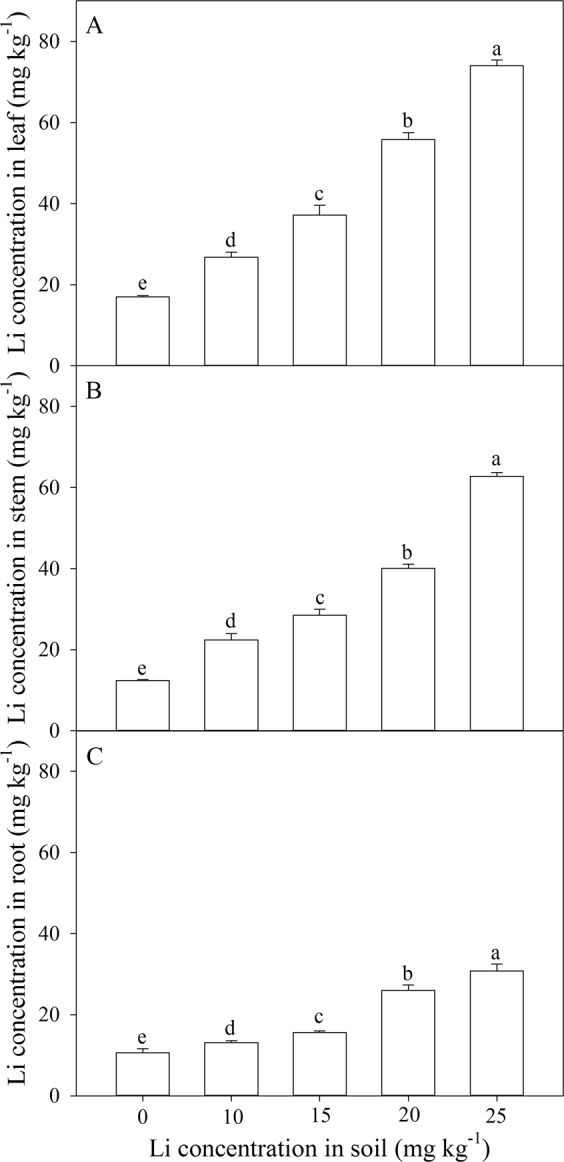
Effect of soil Li addition on TFs concentration in leaf (**A**), stem (**B**) and root (**C**) of *A. venetum*. The values (mean ± S.E) share the same letters above the bars are not significantly different at P < 0.05. n = 5.

Total flavonoids were significantly influenced by different Li treatments (Fig. 3). Among different plant parts, the highest TFs concentration were noted in leaves. Among different Li treatments, the highest value of TFs concentrations in leaves was under 25 mg kg^−1^ soil Li addition. An application of 25 mg kg^−1^ increased 24% TFs concentrations in leaves as compared with control. The TFs concentrations in leaves were not significantly affected by other soil Li treatments (Fig. 3A). TFs concentrations in stems were not significantly affected by soil Li addition (Fig. 3B). The highest value of TFs in roots was under 10 mg kg^−1^ soil Li addition (Fig. 3C).

### Rutin and hyperoside concentration

**Figure 4:**
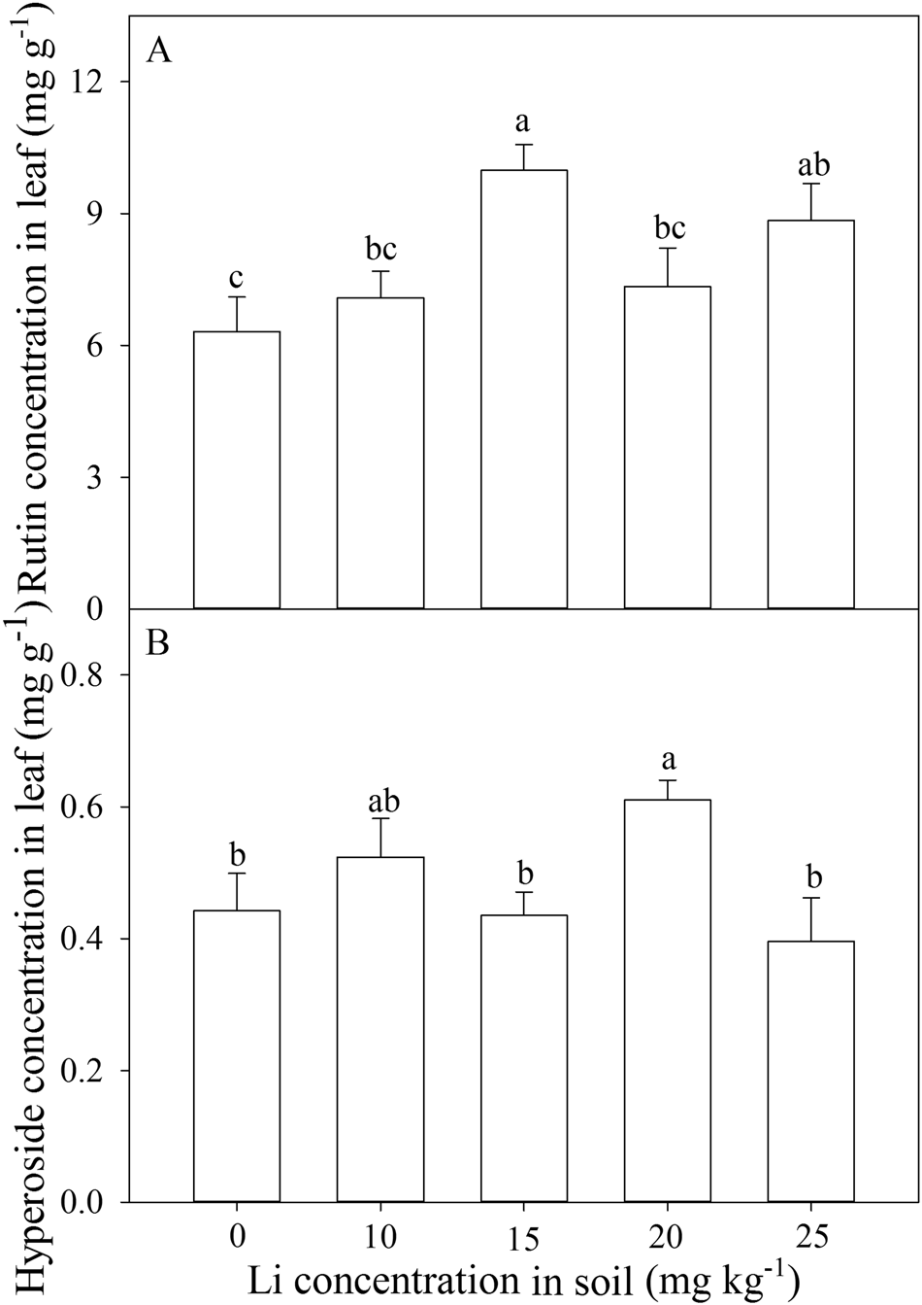
Effect of soil Li addition on rutin (**A**) and hyperoside (**B**) in leaves of *A. venetum*. The values (mean ± S.E) share the same letters above the bars are not significantly different at P < 0.05. n = 5.

The rutin and hyperoside concentrations were significantly influenced by different Li treatments (Fig. 4). An application of 25 mg kg^−1^ Li increased 40% rutin concentration in leaves (Fig. 4A), but did not increase hyperoside concentration in leaves as compared with control (Fig. 4B). Rutin reached the highest value (9.98 mg g^−1^) at 15 mg kg^−1^ Li treatment and hyperoside reached the highest value (0.61 mg g^−1^) at 20 mg kg^−1^ Li (Fig. 4A).

### Antioxidant properties determined by DPPH and FRAP assay

**Figure 5:**
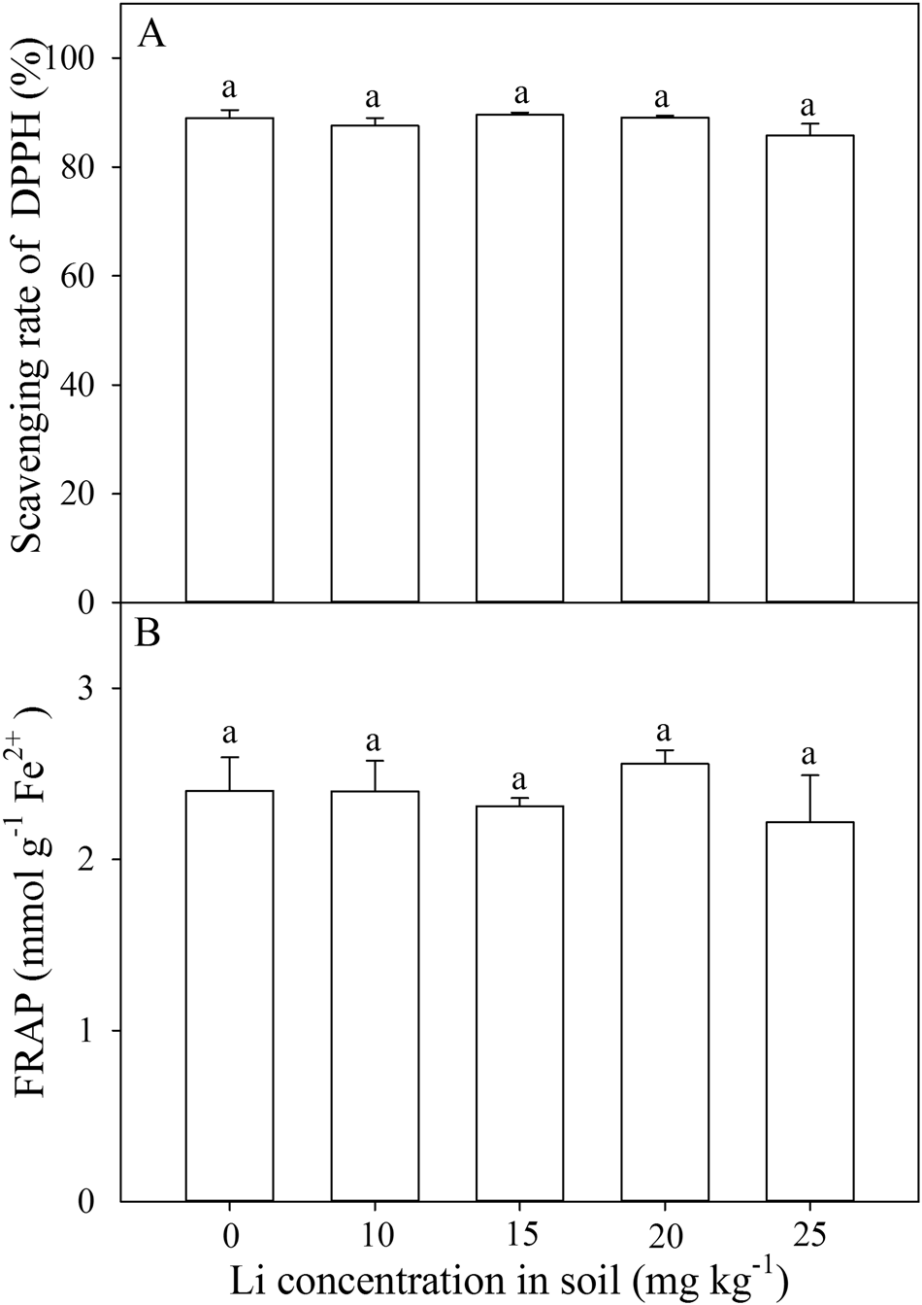
Effect of soil Li addition on antioxidant capacity determined by DPPH (**A**) and FRAP (**B**) assay in leaves of *A. venetum*. The values (mean ± S.E) share the same letters above the bars are not significantly different at P < 0.05. n = 5.

The antioxidant properties determined by the DPPH and FRAP assay were not significantly influenced by different Li treatments (Fig. 5). Among different Li treatments, the scavenging rates of DPPH in leaves were 85.56–89.57%, similar with that of black tea (89.54%), but lower than that of green tea (91.43%) (Fig. 5A). Among different Li treatments, the values of FRAP in leaves was 2.22 mmol g^−1^ Fe^2+^ −2.56 mmol g^−1^ Fe^2+^, similar with that of black tea (2.65 mmol g^−1^ Fe^2+^), but lower than that of green tea (9.15 mmol g^−1^ Fe^2+^) (Fig. 5B).

## Discussion

Although seed germination, plant growth and eco-physiological responses of *A. venetum* to environmental stresses, such as drought and salinity, have been studied extensively^22,23^, these data are the first in which growth promotion effect of Li as a beneficial element to *A. venetum* has been documented. In addition, our data indicated that there was a dose-dependent increase in the accumulation of Li without a decrease in biomoss and TFs concentrations in leaves of this species. The results indicated that Li application via soil addition might be a proper method to produce Li-biofortified Luobuma tea, which may represent an alternative source of Li in human diet.

Though the nature of positive and negtive effects of Li in higher plants is not clear, plant growth stimulation for some species are documented when exposed to low level of Li^8,9^. For example, plant height, first trifoliate fresh weight and leaf area of *Phaseolus vulgaris* increased significantly at a concentration of 4 mg kg^−1^ Li^24^. In maize, it was noted a significant growth-stimulating effect of this metal at a concentration of 5 mg L^−1^ ^25^. In lettuce, 1–2 mmol L^−1^ LiNO_3_ (about 7 and 14 mg L^−1^, respectively) could stimulate plant growth^26^. The growth of *A. venetum* was influenced by Li through the effects on net photosynthesis, which could be explained by its marked impact on the chlorophyll and the photochemical apparatus. Therefore, the beneficial effect of Li application on plant growth still needs further research. Previously we found that *A. venetum* shows non-significant response to low soil Li concentration (50 mg kg^−1^) while exhibits significant reduction at high Li concentrations (100 and 200 mg kg^−1^)^15^. In our study, at 10–15 mg kg^−1^ Li addition, a growth-stimulating effect of Li was noted. At this treatment, not only leaf dry weight but Li concentration in leaves increased significantly.

*A. venetum* has antioxidant, antihypertensive, antianxiety, antidepressant, and hepatoprotective actions^14^. Previous phytochemical investigations revealed that TFs are the major bioactive constituents in the extract of *A. venetum*^14,27^. In our study, Li application did not decrease TFs concentrations in *A. venetum* with the highest concentrations of TFs in leaves under 25 mg kg^−1^ Li. The rutin and hyperoside are the two main functional and bioactive components of TFs in leaves of *A. venetum*^14^. Li treatments did not change or increase rutin and hyperoside concentrations in *A. venetum*. These concentrations in *A. venetum* were similar with that of Shi *et al*.^19^. Previous studies using DPPH assay and our results tested by DPPH and FRAP indicated that *A. venetum* has high antioxidant capacity^28,29^, which were similar with that of common black tea. Li application did not decrease the antioxidant capacity in *A. venetum*. Our results indicated that Li application did not decrease the benefits of this medicinal herb tea.

Despite the fact that Li is not officially considered to be a micronutrient, some authors have suggested provisional recommended intakes set at 1000 μg/day Li for adult and 100 μg/day Li as the minimum human adult Li requirement^7,30^. Some behavioral disorders have been observed in individuals with Li deficiency^1^. Therefore, it has been suggested to the people who are residing in Li-deficient areas to take sufficient and regular Li supplements^1^. Tea is one of the most popular beverages in the world, with various types consumed. Concentrations of Li in different tea samples as a potential dietary source of Li are evaluated. The concentration of Li in the Luobuma tea is >11 mg kg^−1^ and concentrations for other types of tea ranged from 0.02 to 0.6 mg kg^−1^. For Li-biofortified *A. venetum*, the concentration of Li in leaves was 37.20 and 73.98 mg kg^−1^ under 15 and 25 mg kg^−1^ Li addition. According to previous data and calculations, a daily consumption of 10 g common Luobuma tea can give ca. 88 μg Li intake^16^. Consumption of 10 g Li-fortified Luobuma tea obtained from cultivation with 15 and 25 mg kg^−1^ soil Li addition would constitute 29.7% and 59.2% of the provisional recommended dietary daily intake of Li and above the minimum human adult Li requirement. To meet the minimum human adult Li requirement, 3.4 or 1.7 g Luobuma tea obtained from cultivation with 15 or 25 mg kg^−1^ soil Li addition would be enough.

In conclusion, this and previous studies highlight that *A. venetum* should be considered in further application of Li-fortified products, including medicinal herb tea. Furthermore, soil Li addition did not decrease the TFs, rutin and hyperoside concentrations, and the antioxidant capacity. Therefore, Li-biofortified *A. venetum* leaves could be used as Li supplements and potential natural micronutrient. Further studies should focus on the bioavailability of Li from biofortified Luobuma tea made of *A. venetum* leaves and effects of dietary consumption of this type of tea.

## Supplementary information

Dataset 1
